# Australian sea-floor survey data, with images and expert annotations

**DOI:** 10.1038/sdata.2015.57

**Published:** 2015-10-27

**Authors:** Michael Bewley, Ariell Friedman, Renata Ferrari, Nicole Hill, Renae Hovey, Neville Barrett, Oscar Pizarro, Will Figueira, Lisa Meyer, Russ Babcock, Lynda Bellchambers, Maria Byrne, Stefan B. Williams

**Affiliations:** 1 Australian Centre for Field Robotics, The University of Sydney, NSW 2006, Australia; 2 Coastal and Marine Ecosystem Group, School of Biological Sciences, The University of Sydney, NSW 2006, Australia; 3 Institute for Marine and Antarctic Studies, University of Tasmania, Hobart, Tas 7005, Australia; 4 University of Western Australia, Perth WA 6009, Australia; 5 Commonwealth Scientific and Industrial Research Organisation (CSIRO), Australia; 6 Western Australia Department of Fisheries, Perth, WA 6000, Australia

**Keywords:** Ocean sciences, Biodiversity, Coral reefs, Fisheries

## Abstract

This Australian benthic data set (BENTHOZ-2015) consists of an expert-annotated set of georeferenced benthic images and associated sensor data, captured by an autonomous underwater vehicle (AUV) around Australia. This type of data is of interest to marine scientists studying benthic habitats and organisms. AUVs collect georeferenced images over an area with consistent illumination and altitude, and make it possible to generate broad scale, photo-realistic 3D maps. Marine scientists then typically spend several minutes on each of thousands of images, labeling substratum type and biota at a subset of points. Labels from four Australian research groups were combined using the CATAMI classification scheme, a hierarchical classification scheme based on taxonomy and morphology for scoring marine imagery. This data set consists of 407,968 expert labeled points from around the Australian coast, with associated images, geolocation and other sensor data. The robotic surveys that collected this data form part of Australia's Integrated Marine Observing System (IMOS) ongoing benthic monitoring program. There is reuse potential in marine science, robotics, and computer vision research.

## Background & Summary

Less than 0.05% of the global sea floor has been mapped with sonar swath mapping^1^ at high resolution (tens of meters). Coverage at visual resolution (millimeters) using a camera is substantially lower. Visual resolution permits the detailed analysis of benthic taxonomy; however, this requires image capture at an altitude of several meters above the sea floor, typically traveling slower than walking pace. The growing maturity of AUVs has permitted broader and more systematic visual surveys than traditional diver held cameras or towed video sleds (a system whereby a camera on an underwater sled is attached to a ship by a cable, and towed. The resulting images are lower quality than an AUV as the positioning, particularly altitude, is difficult to control precisely). AUVs can operate continuously and precisely at greater depths, with geolocation, sensor data and stereo images captured several times a second. A 3D visual map of the survey area can then be produced from the data. This abundance of data has introduced a new problem for scientists: efficiently extracting and distilling useful information from the raw data.

**Figure 1:**
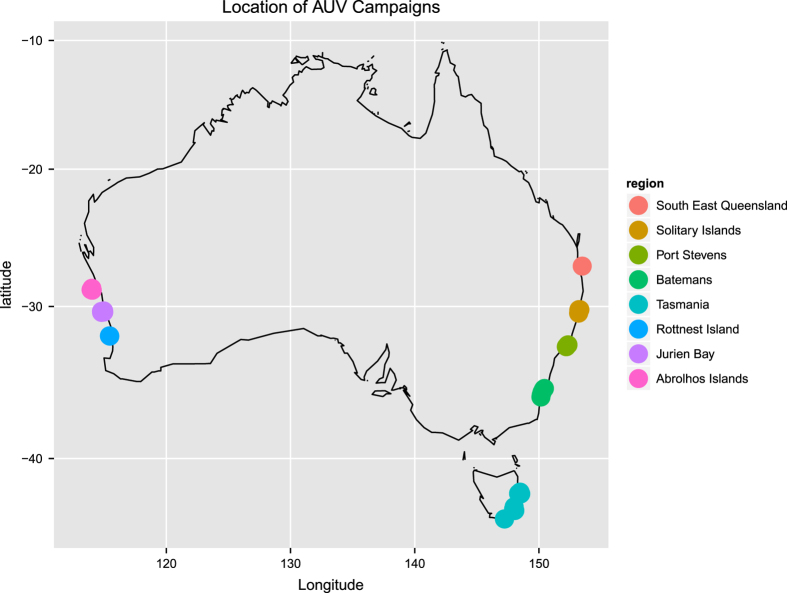
Geographic distribution of annotated images.

**Figure 2:**
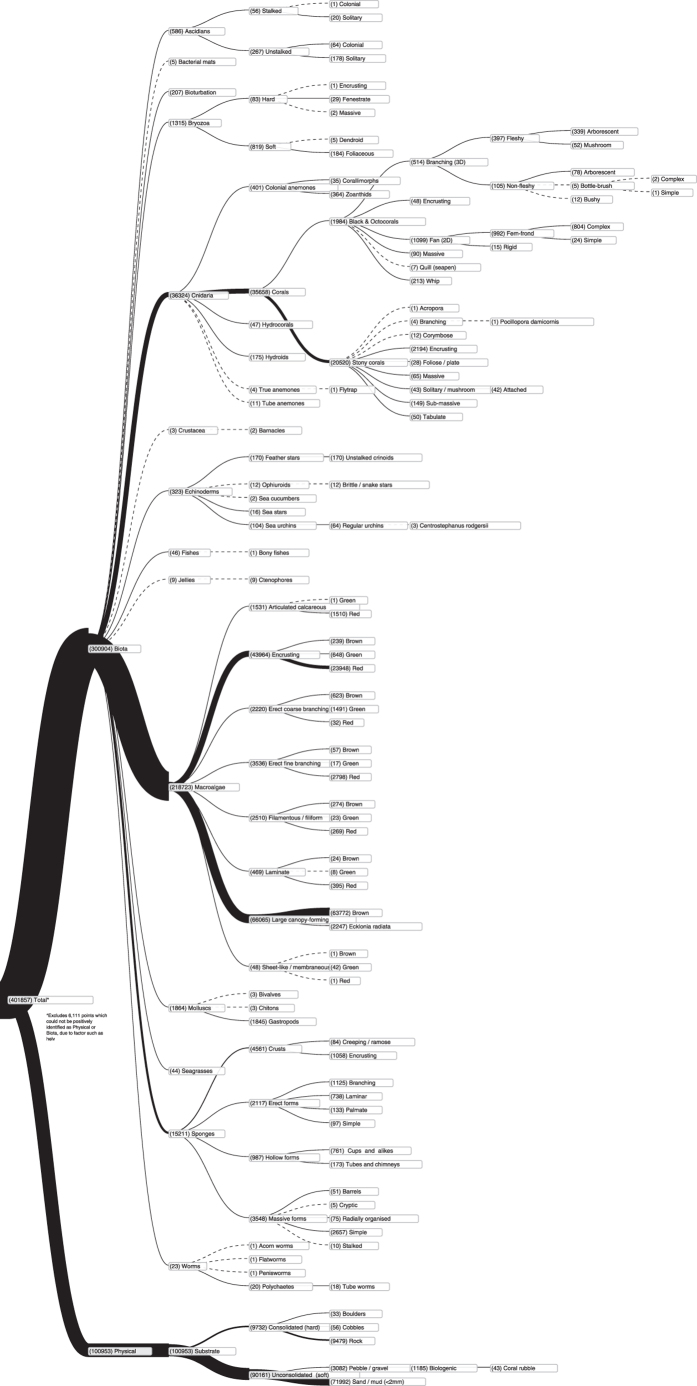
CATAMI Hierarchy diagram. Numbers in brackets show the number of points in the data set that have been labeled as a given class, or one of its descendants. CATAMI Hierarchy has been extended to lower (species) level where appropriate data was available. Best viewed electronically.

The data set presented in this paper contains 407,968 expert annotations of 9,874 georeferenced images with associated sensor data (latitude, longitude, depth, altitude, salinity and temperature) from around the Australian coast (see Fig. 1). The annotations conform to a hierarchy of 148 substratum and biological classes (Fig. 2), and specify the content at specific points within each image. All image and sensor data were captured by the *Sirius* AUV. *Sirius* is the primary platform responsible for collecting seafloor images as part of the AUV facility of the Integrated Marine Observing System (IMOS) in Australia^2^. Table 1 summarizes the number of expert labels applied to each campaign, and Fig. 1 shows the geographic location of each deployment. The annotation process poses a significant bottleneck, taking a trained marine scientist 5 min or more to assign semantic labels to dozens of individual points on a single image using the context provided by the image neighborhood around the point. After a survey is conducted, there is typically a time lag of several years before the labeling is complete, and scientific inferences can begin to be drawn. Even with this delay, it is only practical to label a very small fraction of the data collected by the AUV. For the deployments in this data set, the 9,874 images with labels represent around 2% of the total number of images captured during those deployments.

Machine learning and computer vision techniques have the potential to increase the amount of labeled data and reduce the time it takes to do so. The availability of a set of high quality expert labels with geographic and temporal diversity will permit researchers in these fields to investigate ways to reduce or eliminate the manual labeling effort, as well as gaining new scientific insights from working with a combined data set. Another significant hurdle to the integrated analysis of benthic imagery data is the lack of standardization between research groups. Until recently, individual research groups have labeled images using a variety of custom labeling systems and standards suited to their particular geographic region and research interests, which limits the ability to perform scientific analysis, or train machine learning algorithms on large, varied data sets. In this data set, however, we combine data from four leading research groups, using the recently established Collaborative and Automated Tools for Analysis of Marine Imagery (CATAMI) class hierarchy^3^ as a standardized labeling scheme. The CATAMI scheme permits the various schemes to be combined in a consistent and meaningful way, as shown in Fig. 2.

## Methods

### AUV Data Collection

This data set includes annotated images from an extensive series of AUV-based benthic surveys that were undertaken between 2008 and 2013, around Australia's coastline. Geographic locations include Western Australia, Tasmania, New South Wales and Queensland (Fig. 1). Image and sensor data was gathered by the AUV *Sirius*, as described in ref. 4. The campaigns were conducted by the AUV facility of the IMOS program, funded by the Commonwealth Government and collaborating agencies (see Acknowledgements). During each campaign, *Sirius* executed several missions, deployed at sites selected by the science party, typically focusing on temperate rocky reefs and coral reefs. Missions are defined by a set of georeferenced waypoints and instructions. The AUV autonomously captured images every 0.5 s, while maintaining a nominal 2 m altitude above the sea floor.

The general sampling methodology is described in ref. 4 as being designed to monitor the fundamental reef processes that maintain reef biodiversity and resilience. The processes of interest occur at a number of spatial scales, so a nested hierarchical sampling design was adopted to allow changes to be observed at the differing scales. Deployment mission designs included: (1) Long transects used to monitor broad community structure and integrity, community boundaries, and transitions (2) broad scale, sparse grids on the order of 500–1,000 m on a side to determine spatial variability in habitat structure (3) small-scale 25 *m*×25 *m* full-cover dense grids, providing contiguous coverage mapping for the establishment of long-term monitoring sites. Further detail on target habitat locations, overlapping survey patterns, and other aspects of survey design can be found in ref. 4. Deployments were performed from a ship and typically lasted a few hours, resulting in tens of thousands of stereo image pairs and associated sensor data per dive.

### Expert Annotations

The general approach to annotating images across the four research groups was the same. A subset of images from the dives were selected (e.g. every 100th image), and the commonly used software package Coral Point Count with Excel Extensions (CPCe)^5^ was used to label the content beneath up to 50 uniformly randomly selected points within the image (where the label represents the content under that point, rather than a larger area around it). It should be noted that the data set is therefore unsuited to estimate abundance of rare classes based on individual images; the intended use is to compute statistics over a 25 m×25 m or larger area, or along a transect. Further discussion of marine science literature using up to 50 points per image can be found in the final section of this descriptor.

**Figure 3:**
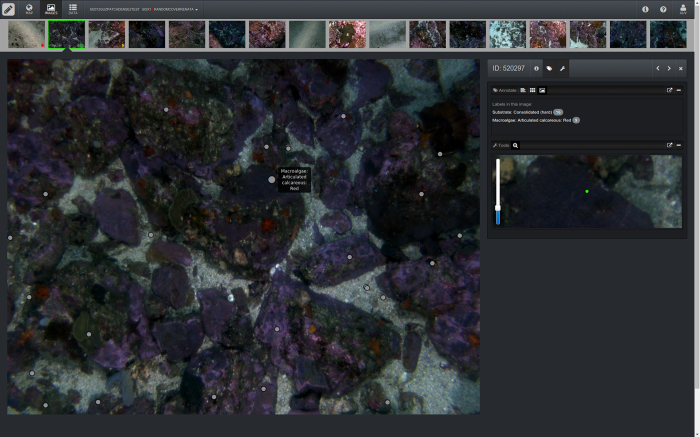
Example of an expert labeled image in *Squidle*.

For the purpose of this data set, the labeling schemes used by the individual research groups were mapped onto the CATAMI hierarchy^3^ (see Fig. 3 for an example). Mapping files used to convert original CPC codes to CATAMI classes were reviewed by the respective marine science groups, and are included as Supplementary files (Data Citation 1), along with a description of each class in the CATAMI hierarchy (Data Citation 1). The following sections describe the differences in methodology between the research groups.

### Western Australia

Images were obtained from three key locations along the Western Australian coastline; Rottnest Island, Jurien Bay and the Houtman Abrolhos Islands. Rocky reef/coral habitat was targeted to establish a series of reference sites for long-term monitoring in the west coast bioregion. Sites were selected based on bathymetry maps and existing knowledge, to target moderate to high relief reef between 15 and 30 m depth. Within each site, 3 replicate grids were surveyed by the AUV which was achieved by conducting a series of parallel, overlapping 25 m long transects, covering a combined area of 625 m^2^ of seabed (i.e. 25×25 m). Replicate grids within a site were positioned approximately 200 m apart. Over 1,000 georeferenced stereo image pairs were collected from each grid. These high resolution images were subsampled at 20 s intervals to generate a sample set of 101–129 non-overlapping images that maximised spatial coverage of each grid^6^. For image analysis, 50 random points were digitally overlaid onto each sample, and the number of points covering each benthic category was counted (using CPCe^5^), then doubled to give a proxy of percent cover. Forty benthic categories, including dominant flora, fauna and substratum characteristics, were determined a priori based on previous research^7^ and used to classify each image. Care was taken to include conspicuous species of considerable ecological importance, such as the canopy-forming brown algae *Ecklonia radiata* and *Scytothalia dorycarpa*, while also using functional or morphological groups to achieve a broad, holistic approach to describing the benthos. Bleached coral was considered ‘alive’, but additional information on the spatial extent of any bleaching was recorded.

### New South Wales

Images obtained during AUV surveys were used to quantify benthic assemblage structure and composition on rocky reefs at three locations along the NSW eastern coastline (Fig. 1). These three locations included highly diverse sub-tropical and temperate rocky reefs between 20 and 50 m depth^8^. Within each location multiple 625 m^2^ dense grids of rocky reef were surveyed at multiple sites using the *Sirius* AUV, sites were at least 1km apart. The AUV achieved full coverage of each 625 m^2^ dense grid (15,000 image pairs), from which 50 spatially balanced images were selected using a generalized random tessellated stratified design in R package *spsurvey*^9^. Each image covered an area of approximately 1.8 m^2^; so, 50 images covered 15% of a 6,625 m^2^ dense grid. Twenty five random points were overlaid on each image and taxa under each point were identified to the highest taxonomic resolution possible using CPCe. The national standard classification scheme CATAMI Version 1.2 was used to identify organisms to a taxonomic, morpho-group (e.g. encrusting coral), major group (Class) and/or morphological level^3^.

### Tasmania

AUV campaigns conducted in 2008 and 2009 targeted reef systems on the Tasman Peninsula on the South-East Coast of Tasmania. AUV transects followed an elongated grid design where the ‘long’ section of transects was oriented down the depth gradient and ‘short’ sections of transect were oriented across the depth gradient. Every 100th image along the transect path (a spacing of approximately 40 m) from one camera was scored using CPCe. In preliminary analyses on the Tasman Peninsula, a spacing of 40 m meant that images generally occurred in the next patch of substratum along the transect and the range of substrata and the values of multibeam derived variables sampled in images was representative of that found in the entire study region. The substratum or biota underneath 50 random points within an image was scored. Benthos was identified to the lowest possible taxonomic or morphological unit using refs 10 and 11. For most sessile invertebrates this was morphospecies, identified by morphology and color. Representative algae were identified to species, otherwise to functional groups, and mobile invertebrates (infrequently observed) were assigned to broad categories (e.g. starfish, sea urchin, mollusc). This scoring approach pre-dates the CATAMI classification scheme, and contains a number of highly specific classes (at species level). For the purpose of this data set, labels were mapped post hoc to the scheme, where the scored class was matched to the deepest valid level of the hierarchy.

### Queensland

The AUV campaign conducted in Queensland in 2010 focused on reef systems east of Moreton Island in southeast Queensland. The AUV mission was intended to cover the full depth range of the reef at Henderson’s South which was approximately 12 m in depth at its shallowest to over 45 m at its greatest depth. A gradation in habitat types was known to occur at this site, transitioning from turf algae and corals in the shallower parts of the reef to kelp forest in the deeper areas. For this reason a regular sub-sampling was undertaken, allowing for the mapping of the spatial features of habitat structure. Transects were located between the depths of 17–42 m on a 400×500 m rectangular grid design with intersecting lines spaced every 100 m. The grid was oriented so that there were five lines roughly E-W perpendicular to depth contours and six N-S roughly parallel to depth contours. From each of these lines 100 m transects were sub-sampled, along the side of each of the cells outlined by the square grid. Ten images were selected from each transect at a spacing of 10 m. The substratum or biota underneath 20 random points within an image was scored using CPCe. Benthic biota were identified to the lowest possible taxonomic or morphological unit using the CATAMI classification scheme. For most sessile invertebrates this was morphospecies, differentiated by morphology and color. Representative algae were identified to species, otherwise to functional groups, and mobile invertebrates (infrequently observed) were assigned to broad categories (e.g. starfish, sea urchin, mollusc).

### Code availability

The production and processing of this data relied on a complex software pipeline, involving controlling a hover-cable AUV, extracting and post-processing the data to produce accurate georeferencing via a Simultaneous Localization and Mapping (SLAM) algorithm, using CPCe to annotate images, and further scripts to import data into the *Squidle* benthic imagery web application. The code for *Squidle*, along with the import scripts, are available on github at http://github.com/acfrmarine/squidle. The script used to import the cpc files into the squidle database is located in that repository at scripts/annotation-scripts/import_cpc_file.py. The most complete descriptions of the data acquisition process are in ref. 12 for image processing, and ref. 13 for navigation and SLAM.

## Data Records

The complete data set described here has been made available on *Squidle* at http://squidle.acfr.usyd.edu.au. *Squidle* is a new web-based framework that facilitates the exploration, management and annotation of marine imagery. It provides a user-friendly interface that integrates spatial map-based data management tools with an advanced annotation system. The online annotation system permits scientists to easily collaborate on both the labeling and use of their data. It will in future also provide a platform for using and testing machine learning and computer vision algorithms on marine imagery. This data set has been made available to view, explore and download via the web interface. Most of the expert annotations were produced prior to the development of *Squidle* and the CATAMI scheme, and have been imported into *Squidle* CPCe. By comparison, CPCe is a standalone application for individual users to label marine images locally, and does not include higher level features such as data exploration and online collaboration. A comparison between *Squidle*, CPCe, and other platforms was performed in ref. 14.

It should be noted that the although the *Squidle* platform is the easiest way to explore the data set, it is still under active development. An image downloader tool is available on the site, as well as the ability to download non-image data as csv files. In addition to *Squidle*, the data set is also available from a number of other sources.

A table of expert annotations, sensor data, geolocation and image metadata for BENTHOZ-2015 is available for download from *figshare* (Data Citation 1), the online scientific data repository.

All images and sensor data (without expert annotations) captured by the AUV *Sirius* are available on the Australian Ocean Data Network (AODN) web portal at http://imos.aodn.org.au (with data available for direct download from http://data.aodn.org.au/IMOS/public/AUV/). The AODN Portal is the official repository for IMOS AUV survey data; as such it contains images and sensor data from a large number of surveys not included in this data set. Note that there is no mechanism to select the precise set of data defined by BENTHOZ-2015.

### Expert Labels

The expert labels are available as a comma separated value file (Data Citation 1), where each row represents a single expert labeled point within an image. The fields are described in Table 2 (available online only), and consist of a unique identifier, the image containing the point, and the location of the point within that image.

**Figure 4:**
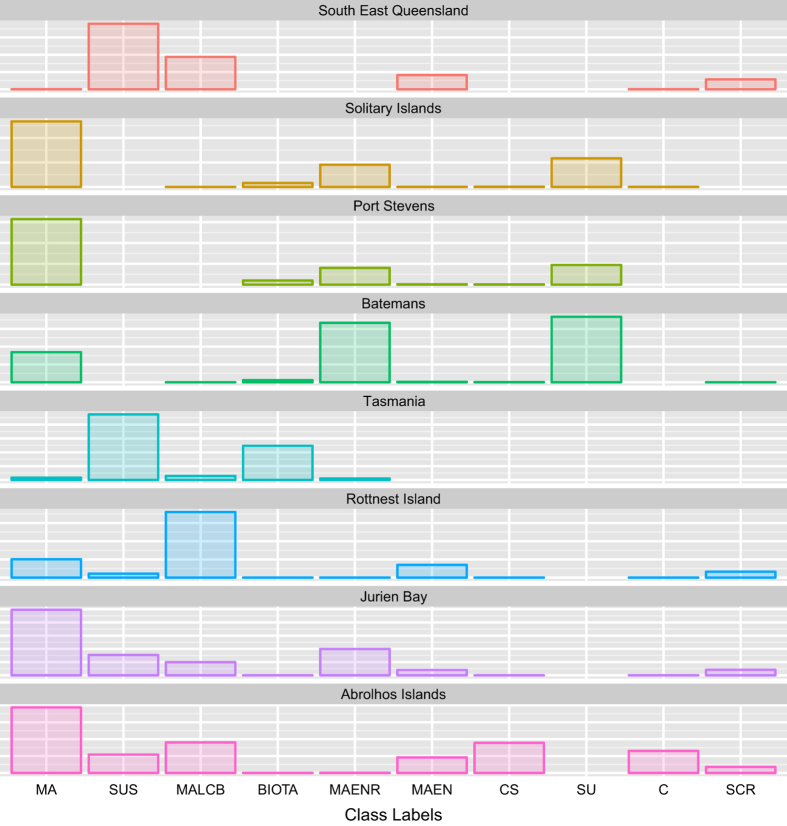
Frequencies of most popular class labels appearing in each region.

The ‘label’ field defines the class within the CATAMI hierarchy that has been assigned to each point. Figure 4 shows the frequency of the most popular class labels appearing in each region, after mapping to the CATAMI hierarchy. Note the heterogeneity in the labeling with, for example, *SUS* (sandy substrate) dominating in Tasmania, and *MA*(Macro Algae) dominating in Western Australia. These differences should not be solely attributed to differences in biogeography, due to the deliberate non-random selection of deployment sites with different scientific aims (the only true random samples in the data set are the selection of N points within each annotated image). Each label in the hierarchy corresponds to a *Codes for Australian Aquatic Biota* (CAAB) Code, which acts as an Australia-wide identifier for aquatic organisms. The codes are described in more detail at http://www.marine.csiro.au/caab/.

It is also important to note that some of this heterogeneity is lower than initially appears—e.g. *MAENR* (Macro Algae Encrusting Red) is a more specific type of *MA*. This variation in specificity of labels is driven by research groups' areas of expertise, and scientific interests.

### Image Metadata

**Figure 5:**
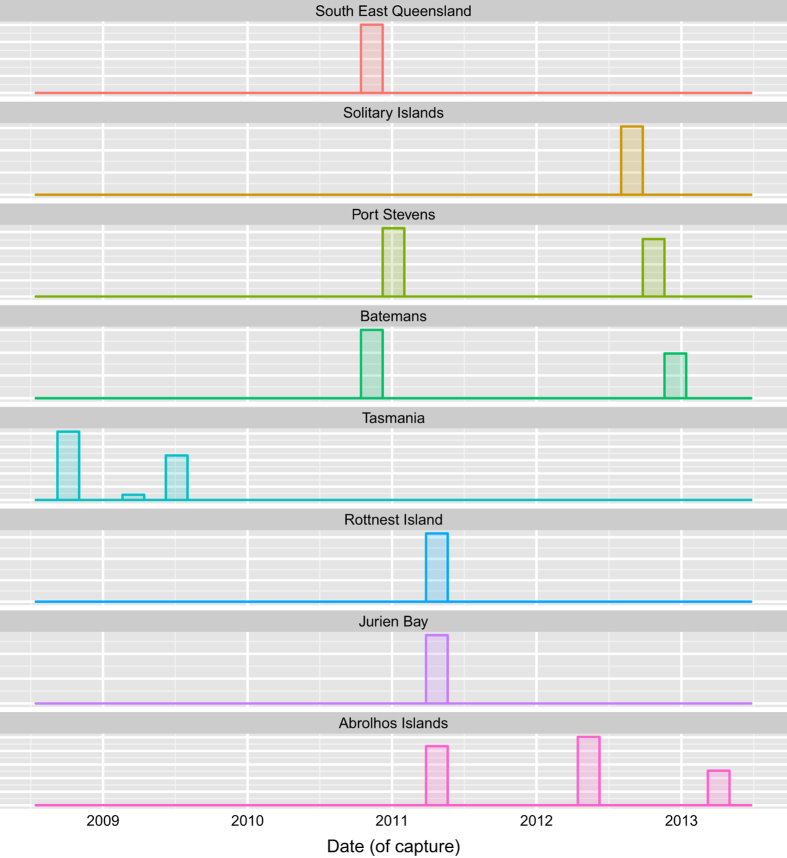
Distribution of annotated images over time.

**Figure 6:**
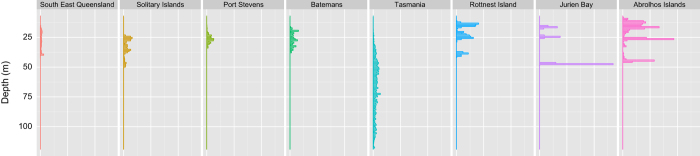
Distribution of annotated images over depth.

As the AUV captures an image at a particular point in time, other metadata can be assigned to that image, such as vehicle position, and additional sensor data being recorded by the AUV. These fields are described in Table 3 (available online only). Figs 5 and 6 show the time and depth distributions over which the annotated images were captured in each region. The precise georeferencing represents a significant advantage of using an AUV based data set, aside from the sheer volume of data collected. Repeat surveys of the same areas (some of which are present in this data set) can be compared to accurately evaluate changes over time^15,16^.

### Images

Images are downloadable as PNG files with lossless compression, typically between 1.3 and 2.4 MB per image. Some simple batch processing was performed to enhance the images, described in ref. 12. Because of the large size of the data set (approximately 10,000 annotated images, or two orders of magnitude larger including unlabeled ones), images are made available using a separate downloader tool. Users are requested to download only the data they intend to use. *Squidle* and the IMOS AODN Portal also permit exploration of the images using a web browser without requiring a bulk download. Image acquisition is described in detail in ref. 12, using a stereo camera pair. The color camera was used to capture the images used here, which has an approximate field of view of 42×34 degrees, for the 1,360×1,024 pixel RGB images. At a typical altitude of 2 m, this corresponds to an image approximately 1.5 m by 1.2 m, with an area of approximately 1.8 m^2^, and pixels representing approximately 1 mm in extent. The non-flat nature of the sea floor, and camera geometry, mean that these measurements should not be considered precise. The altitude of each image is provided in the data set, so some spatial scaling can be made. The roll, pitch and yaw of the camera is approximately fixed (pointing downward), due to the passively stable design of the AUV *Sirius*.

### Data set definitions

In order to support the needs of machine learning researchers, a separate ‘secret’ test set has been reserved (not included in the numbers and figures in this paper) as (Data Citation 1). This allows predictive models to be developed using the publicly available training data set described here, and then tested against a previously unseen set of test data. The test data set consists of a small number of individual deployments (representing particular geographic locations) that were selected across geographic location and year. All labeled points on those images are available, but listed as ‘Unknown’ class in the publicly downloadable data. The training set and test set are both available separately for download from *Squidle* and figshare.

The training set has been organized into the following hierarchy:

- A *campaign* consists of a series of deployments conducted on a single field trip in a geographic area (e.g Tasmania 2008).
- Each campaign is broken into a series of *deployments*, each of which represents a continuous set of data starting when *Sirius* was launched from the support vessel, until *Sirius* was recovered, usually several hours later.

## Technical Validation

Annotation data in this data set were produced by a combination of experienced scientists and trained research students, all scorers having either considerable prior experience in benthic image annotation or extensive training in both benthic image annotation and the used classification scheme. Selection of interested students was undertaken before training so that only the most committed and skillful students with suitable underwater experience were invited to participate. All students underwent the same training, with individual supervision and help with taxa identification. Training lasted between 1 and 4 weeks depending on the student skill and previous experience. Annotations generated during training were not added to the database. The authors trained all non-experts who contributed data to this data set. Quality control of labels varied slightly for each state, as described below. The typical usage of expert annotations in the literature does not take into account a quantification of the label accuracy. There is no practical means by which to obtain a ‘gold standard’ reference (as the biota and physical morphology changes over time, and precise georeferencing is difficult. Instead, the most suitable means is to compare inter-expert agreement on point labels based on the same set of images. With a data set of this complexity (148 classes, and approximately 10,000 images from a diversity of geographic regions), this validation is best designed and performed with a particular application in mind, and does not require any information other than that contained in this data set and descriptor. Researchers making use of this data set are invited to assess the validity of the data set for their purposes.

### Western Australia

A total of 7 people labeled this data set, from which 3 were experts and 4 trained non-expert researchers. Consistency among the 7 labelers was verified by having everyone score the same points in the same images. Emphasis was placed on correct identification of broad groups (algae, coral, sponges, other sessile invertebrates and seagrass), and accurate identification of algal and coral morphotypes (i.e. fleshy vs calcareous, red vs brown or green, plating vs massive coral). A photo identification guide was created and constantly updated and re-circulated to all labelers. Any uncertain labels were flagged and verified by one of the 3 experts. When comparing expert labeled points to trained labeled points, the only difference was that experts tended to label shadowed points with taxa, while trained labelers would classify those as points as uncertain, and redirect them to experts.

### New South Wales

A total of 5 people labeled this data set, from which one was an expert and 4 trained non-expert research students. Consistency among the 5 labelers were verified by having everyone score the same points in the same images. Annotations between trainer and students were carefully compared and students only started annotating after consistency was at least 85% at the morpho-taxa level for all biotic and abiotic groups (e.g. fine branching fleshy red algae). This threshold was typically achieved after 2–3 weeks of intensive training. A photo identification guide was created and constantly updated and re-circulated to all labelers. Any uncertain labels were flagged and verified by the expert.

### Tasmania

A total of 3 experts labeled this data set, from which 1 coordinated an image catalog and morpho-species names to avoid duplication. Initially, the coordinator scanned through the imagery and identified and cataloged many of the conspicuous and common taxa. Regular meetings (i.e. about every second day) were held during the scoring process to go through any new species observed to ensure consistency of labeling, and for the labeling process itself. The coordinator randomly checked images during the scoring process and again at the end to check that scoring was consistent between and within scorers. Any systematic issues found were addressed at this stage by re-scoring points with errors.

### Queensland

A total of two people labeled this data set, from which one was an expert and one trained non-expert research students. Initially, both the scorer and an expert examined 40 images simultaneously for consistency in labeling. During the entire labeling process any uncertain labels were flagged and verified by the expert. At the end of the process data were plotted and checked for outliers, with any outliers checked by the expert and re-scored if necessary.

### Geolocation Accuracy

The positional accuracy of the AUV is described in ref. 17. Based on the sensors (including GPS on surface for an initial position fix with ship borne ultra-short baseline (USBL) positioning underwater), the accuracy is approximately ±5 m. The authors also describe several techniques based on visual feature matching that permit this to be reduced when comparing multi-year surveys in the same locations.

## Usage Notes

### Automated Labeling Research

The primary motivation for creating and releasing this data set was to enable progress towards automated labeling solutions for benthic imagery. In terms of supervised machine learning, the labels can be considered an example of a hierarchical classification problem^18^. A particular set of algorithms and approaches can be applied to automatically recognizing content within a taxonomy such as CATAMI. Alternatively, the problem can be recast as a single binary classification task (such as presence or absence of a particular class), or mutually exclusive set of classes (such as algae, coral, sponges and ‘other’).

Further, the points can be aggregated at an image level, in order to train and predict on overall habitat composition of images. Unsupervised techniques could also be used on the images to automatically detect visually similar groups of images.

A comprehensive review of the benthic computer vision literature up to 2012 can be found in ref. 19 (in which Table 2.1 contains references to work focusing on classifying whole images, and Table 2.2 covers point level classification in images). Since then, other relevant work includes^20–23^. The latter two look at the Tasmania 2008 campaign from this data set, with^23^ performing hierarchical classification using a subset of the CATAMI hierarchy.

### Geographical and Temporal Variation

One aim of this data set is to permit researchers to examine the robustness of their techniques to temporal and geographic variation. In terms of temporal variation, the Abrolhos region of Western Australia has data for dives in a small geographical area, from 2011, 2012 and 2013. Similarly, there is data from repeated surveys in New South Wales and Tasmania. In terms of geographic variation, the data set includes locations from a wide diversity of locations around the Australian coast. Common species such as *Ecklonia radiata* (kelp) are known to vary greatly in appearance between tropical and temperate areas, making for a very challenging machine learning problem. Aside from the sheer number of labels, this variety is the most unique feature of the data set. Without this type of collaborative data set, machine learning and computer vision researchers are typically restricted to working with a single data set from a particular bioregion, and making untestable assumptions about their algorithms’ ability to generalize.

### Image Processing

As the AUV platform and survey process has matured over the years, changes have been made both on board *Sirius*, and in the post-processing of the data. Xenon strobes, for example, were replaced with white LEDs. The processed images represent the best processing available at the time the campaign was conducted, and importantly the processing that was used by experts when labeling the images. The raw images can also be made available by special request, for those researchers wishing to investigate their own image processing techniques. The post-processing steps are described in ref. 12.

### Testing Performance

In order to facilitate easy and fair comparison of different approaches, a hidden test set (described earlier) has been reserved. Researchers wishing to publish performance results on the test set are encouraged to contact the authors.

### Marine Science

Aside from automated labeling research, this data set also has potential value for direct scientific insights. By making a wide variety of label data available in a standard format, studies involving a comparison across time or geographic location become much more feasible, such as the recent study of *Ecklonia radiata* distribution around Australia^24^. Rather than solely comparing conclusions drawn by various studies at a high level (as a meta-study), the entire set of raw data is available for analysis as a single group.

Other marine science studies using similar types of data (up to 50 expert labeled points per benthic image) include^25–28^. One highly influential work used average abundances over large areas to demonstrate a 27 year decline of coral cover on the Australian Great Barrier Reef^29^.

## Additional information

**How to cite this article:** Bewley, M. *et al.* Australian sea-floor survey data, with images and expert annotations. *Sci. Data* 2:150057 doi: 10.1038/sdata.2015.57 (2015).

## Supplementary Material



## Figures and Tables

**Table 1:** Data set regional summary.

**Geographic Area**	**Survey Year**	**# Labels**	**# Images**
Abrolhos Islands	2011, 2012, 2013	119,273	2,377
Tasmania	2008, 2009	88,900	1,778
Rottnest Island	2011	63,600	1,272
Jurien Bay	2011	55,050	1,101
Solitary Islands	2012	30,700	1,228
Batemans Bay	2010, 2012	24,825	993
Port Stevens	2010, 2012	15,600	624
South East Queensland	2010	10,020	501
The whole data set contains 407,968 expert labelled points, on 9,874 distinct images.			

**Table 2:** Expert label fields

**Data field**	**Description**	**Values**
*kpid*	A unique identifier for an expert labeled point in an image	string
*image__id*	A unique identifier for the image this point applies to	integer
*y*	Fraction of the point from the top of the image	numeric (0–1)
*x*	Fraction of the point position from the left of the image	numeric (0–1)
*label*	A unique number assigned to the point	integer
*code*	An abbreviation of the class name assigned to the point	string

**Table 3:** Image metadata fields

**Data field**	**Description**	**Values**
*image_name*	The unique identifier of an image (1360x1024 pixels, RGB)	string (no file extension)
*date_time*	Time stamp of image, in UTC	string (YYYY-MM-DD HH:mm:ss+00:00)
*campaign*	The campaign during which the image was captured	*string (<region> YYYY)*
*deployment*	The name of the deployment, within the campaign	string (rYYYYMMDD_HHmmss_<name>
*latitude*	The latitude of the vehicle	Decimal degrees
*longitude*	The longitude of the vehicle	Decimal degrees
*depth*	The depth from the surface in metres of the camera	Positive numeric (More positive is deeper, in metres)
*altitude*	The height of the camera above the sea floor, according to the Doppler Velocity Log	Positive numeric (m)
*salinity*	The salinity measured by the vehicle	Numeric (psu)
*temperature*	The temperature of the water measured by the vehicle	Numeric (Celsius)
