# Corrigendum: Australian sea-floor survey data, with images and expert annotations

**DOI:** 10.1038/sdata.2016.113

**Published:** 2016-12-20

**Authors:** Michael Bewley, Ariell Friedman, Renata Ferrari, Nicole Hill, Renae Hovey, Neville Barrett, Ezequiel M. Marzinelli, Oscar Pizarro, Will Figueira, Lisa Meyer, Russ Babcock, Lynda Bellchambers, Maria Byrne, Stefan B. Williams

**Keywords:** Ocean sciences, Biodiversity, Coral reefs, Fisheries

*Scientific Data* 2:150057 doi:10.1038/sdata.2015.57 (2015); Published 27 Oct 2015; Updated 20 Dec 2016

The authors regret that Ezequiel Marzinelli was omitted in error from the author list of the original version of this Data Descriptor. This omission has now been corrected in the HTML and PDF versions of this Data Descriptor. In addition, support from the Australian Research Council was not acknowledged in the original version of this Data Descriptor, and the authors would like to thank them for support now.

